# Restitution slope is principally determined by steady-state action potential duration

**DOI:** 10.1093/cvr/cvx063

**Published:** 2017-03-23

**Authors:** Michael J. Shattock, Kyung Chan Park, Hsiang-Yu Yang, Angela W. C. Lee, Steven Niederer, Kenneth T. MacLeod, James Winter

**Affiliations:** 1 Cardiovascular Division, King’s College London, St Thomas’ Hospital, Westminster Bridge Road, London SE1 7EH, UK;; 2 NHLI, ICTEM Building, Hammersmith Campus, Du Cane Road, London W12 0NN, UK;; 3 Department of Surgery, Division of Cardiovascular Surgery, Tri-Service General Hospital, National Defense Medical Center, Taipei 114, Taiwan;; 4 Biomedical Engineering, King’s College London, St Thomas’ Hospital, Westminster Bridge Road, London SE1 7EH, UK

**Keywords:** Action potential duration, Electrical restitution, Restitution, Cardiac memory

## Abstract

**Aims:**

The steepness of the action potential duration (APD) restitution curve and local tissue refractoriness are both thought to play important roles in arrhythmogenesis. Despite this, there has been little recognition of the apparent association between steady-state APD and the slope of the restitution curve. The objective of this study was to test the hypothesis that restitution slope is determined by APD and to examine the relationship between restitution slope, refractoriness and susceptibility to VF.

**Methods and results:**

Experiments were conducted in isolated hearts and ventricular myocytes from adult guinea pigs and rabbits. Restitution curves were measured under control conditions and following intervention to prolong (clofilium, veratridine, bretylium, low [Ca]e, chronic transverse aortic constriction) or shorten (catecholamines, rapid pacing) ventricular APD. Despite markedly differing mechanisms of action, all interventions that prolonged the action potential led to a steepening of the restitution curve (and vice versa). Normalizing the restitution curve as a % of steady-state APD abolished the difference in restitution curves with all interventions. Effects on restitution were preserved when APD was modulated by current injection in myocytes pre-treated with the calcium chelator BAPTA-AM – to abolish the intracellular calcium transient. The non-linear relation between APD and the rate of repolarization of the action potential is shown to underpin the common influence of APD on the slope of the restitution curve. Susceptibility to VF was found to parallel changes in APD/refractoriness, rather than restitution slope.

**Conclusion(s):**

Steady-state APD is the principal determinant of the slope of the ventricular electrical restitution curve. In the absence of post-repolarization refractoriness, factors that prolong the action potential would be expected to steepen the restitution curve. However, concomitant changes in tissue refractoriness act to reduce susceptibility to sustained VF. Dependence on steady-state APD may contribute to the failure of restitution slope to predict sudden cardiac death.

## 1. Introduction

Action potential duration (APD) restitution, the process of rate-dependent adaptation of the cardiac action potential, has been proposed as a mechanistic determinant of the stability of re-entrant arrhythmia. The original restitution hypothesis, first described in 1968 by Nolasco and Dahlen, suggests that steep restitution relationship will promote ventricular fibrillation (VF).^1^^,^^2^ In this model, a slope >1 acts to amplify oscillations in APD (alternans) resulting in conduction block and wavebreak. Flattening of the electrical restitution curve—that results in a gradual decrease in alternans towards a steady-state APD—was proposed as a potential target for anti-arrhythmic therapy. However, subsequent research showed that the steepness of the restitution curve is not simply a function of the DI, but also of the previous pacing history (i.e. of short-term ‘cardiac memory’), and, therefore, different values for restitution slope are generated by different pacing protocols.^3^ It was also recognized that single-site measures of restitution slope fail to account for spatial electrophysiological heterogeneities and the impact of conduction velocity restitution at short DIs.^4^ A general theory of the mechanism VF maintenance has been proposed; where the interaction of APD and conduction velocity restitution acts to sustain a pattern of wavebreak and self-regenerating re-entry.^5^ However, there is also substantive evidence that VF cycle length (CL), and therefore VF maintenance, is largely determined by local tissue refractoriness.^6–8^

Although the restitution hypothesis is likely to be incorrect—at least in its original form—several other theories have implicated APD-restitution in arrhythmogenesis. Firstly, restitution is thought to be critical for functional re-entry in conditions of increased dispersion of repolarization, where a steep restitution relationship in the region of proximal to the line of conduction block is essential for ‘successful’ re-entry.^9^^,^^10^ Secondly, modelling studies suggest that spatial heterogeneity of restitution slope, in conjunction with premature stimuli, can generate a substrate that favours re-entry and, interestingly, proposed electrocardiographic measures of ‘restitution heterogeneity’ have been shown to predict patients at high risk of ventricular arrhythmia.^11^^,^^12^

Typically, the restitution curve is fitted to a mono-exponential function, such as that shown in Eq. (1).

Equation 1 $$\text{APD}={\text{APD}}_{\text{ss}}*[1-b*\;\text{exp}(-\text{DI}/\tau )]$$

Accordingly, the slope of the restitution curve is determined by four factors; the maximum, or steady-state, action potential duration (APD_ss_), the minimum DI, the time constant (τ) and the normalized minimum APD (b). An increase in APD_ss_ or b, decrease in τ, or shorter minimum DI would be associated with a steeper curve. However, the relative importance of each parameter in determining the restitution characteristics of the mammalian ventricle has not been extensively studied. Review of the literature indicates that there is a strong association between APD_ss_ and the steepness of the electrical restitution curve.^13–20^ Despite this, a mechanism relating APD_ss_ and restitution slope has not been described, and the importance of APD_ss_ as a determinant of restitution kinetics is largely unappreciated. Critically, if a mechanistic link can be shown, this has implications for theories on the mechanisms of re-entrant arrhythmia, because the duration of the cardiac action potential also determines tissue refractoriness.

Recent experimental studies have highlighted the importance of APD_ss_ as a determinant of the rate-dependent properties of the ventricular myocardium. The geometric relation between APD and repolarization rate (or net ionic current) has been proposed as a mechanistic basis for the reverse rate-dependent action of many class III anti-arrhythmic drugs.^21–24^ Studies by Bányász et al. and Bárándi et al. demonstrate the importance of this relationship in steady-state adaptation of the ventricular action potential at long CLs (300–5000 ms), in a range of species.^22^^,^^23^ In principle, the same mechanism may be an important determinant of the slope of the restitution curve, as we recently proposed.^25^ However, some experimental evidence is contrary to this claim, as stimulation of the vagus nerve, and treatment with the anti-arrhythmic agents bretylium and ibutilide, have been reported to prolong APD but to flatten the relationship between APD and DI.^1^^,^^26^^,^^27^ This raises the question of whether the dynamics of APD-restitution and steady-state APD can or cannot be dissociated? That is to say, can restitution slope change independently of APD. Clinically this may be important; (i) because of the potential contribution of the APD-restitution curve and regional restitution characteristics to the electrical stability of the myocardium and (ii) because alterations in APD and dispersion of APD are a common consequences of cardiac conditions such as acute ischaemia, heart failure and long QT syndrome. Moreover, many therapeutic drugs exert effects on APD, either by design, as with class III antiarrhythmic agents, or because of off-target effects. In the present study, we examined the relationship between APD, the steepness of the electrical restitution curve and ventricular refractoriness. We aimed to test the hypothesis that restitution slope is principally determined by APD_ss_ and that, as a consequence, restitution slope will change concomitantly with tissue refractoriness.

## 2. Methods

### 2.1 Animal welfare

All procedures were undertaken in accordance with ethical guidelines set out by the UK Animals (Scientific Procedures) Act 1986 and Directive 2010/63/EU of the European Parliament on the protection of animals used for scientific purposes. Studies conformed the Guide for the Care and Use of Laboratory Animals published by the U.S. National Institutes of Health under assurance number A5634-01. Studies were approved by local ethics review at King’s College London.

### 2.2 Isolated heart studies

Studies were performed in male adult Dunkin–Hartley guinea pigs (450–650 g) and New Zealand White Rabbits (3.5–4.5 kg). The innervated rabbit heart preparation was isolated as previously described.^28^ All animals were euthanized by sodium pentobarbitone overdose (160 mg/kg, i.p. or i.v.,) with concomitant heparin (100–1000 units). Isolated hearts were perfused in Langendorff mode with an oxygenated (95% O_2/_5% CO_2_) Krebs–Hensleit buffer solution containing (in mmol/L): NaCl 114, KCl 4, CaCl_2_ 1.6, NaHCO_3_ 24, MgSO_4_ 1, NaH_2_PO_4_ 1.1, glucose 11.0 and sodium pyruvate 1.0. Hearts were instrumented for the recording of left-ventricular mono-phasic action potentials (MAP), using a Franz-type recording electrode (electrode spacing = 2 mm, electrode diameter = 1 mm). Hearts were paced at twice the diastolic threshold (2-ms pulse duration) from the right ventricular endocardial apex.

#### 2.2.1 Restitution protocols

Initial experiments investigated changes in the dynamic restitution curve in guinea pig hearts following inhibition of the delayed rectifying potassium currents (clofilium tosylate, 150 nmol/L in DMSO *n* = 7), activation of the inward sodium current (veratridine, 300 nmol/L in DMSO *n* = 7), activation of the l-type calcium current (Bay-K 8644 (±), 500 nmol/L), with reduced extracellular Ca ([Ca]_e_, 1.0 mmol/L *n* = 7) and with catecholamine perfusion (100 nmol/L noradrenaline + 25 nmol/L adrenaline + 50 μmol/L ascorbic acid *n* = 7). Final DMSO concentration was <0.001% (v/v) in all groups. Each heart acted as its own control. For assessment of the dynamic restitution curve, hearts were paced at an initial CL of 250–200 ms (depending on the intrinsic rate) for a period of 50 beats. Pacing CL was then reduced by 25 ms, and hearts were paced for a further 50 beats. This process was repeated until loss of capture occurred or VF was induced.

The dependence of the restitution curve on its preceding pacing history was investigated by the standard extra-stimulus (S1–S2) restitution protocol. Hearts (*n *= 10) were paced at an initial CL of 200 or 170 ms for a period of 2 min to allow for APD to adapt to the underlying rate. Extra-stimulus restitution curves were subsequently generated by pacing with a 20-beat drive train (200 or 170 ms CL, S1) followed by an extra-stimulus (S2) delivered at progressively shorter coupling intervals.

Additional experiments investigated the effects of electrical stimulation of the right cervical vagus nerve on restitution relationships in the isolated innervated rabbit heart. Nerves were isolated and stimulated as previously described.^28^ Dynamic restitution relationships were measured at baseline and during vagus nerve stimulation at 10 or 50 Hz (5 V, 2-ms pulse duration). Hearts were paced at a fixed CL (220 ms) to normalize for the influence of heart rate. Pacing CL was decreased 10-ms steps, at 50-beat intervals, until 2:1 block or VF induction. Nerve stimulation was maintained throughout the dynamic pacing protocol.

#### 2.2.2 VF susceptibility

Further studies aimed to assess the relationship between changes in APD, restitution slope and vulnerability to sustained VF. Studies were conducted in isolated guinea pig hearts with intact autonomic innervation. Animals were randomized to 1 of 4 interventions: (i) ibutilide (100 nmol/L), (ii) flecainide (3 μmol/L), (iii) sympathetic nerve stimulation (SNS, bilateral, 5 V 3–4 Hz) and (iv) ischaemia (5 min at 30% flow with pacing at 5 Hz).

Due to the need to resolve both pro- and anti-fibrillatory effects on VF susceptibility, two burst pacing protocols were utilized; a 10×30 ms CL burst, with low baseline incidence, and a 60×30 ms CL burst, with higher baseline incidence. Protocols were repeated 4× in each heart, and susceptibility was assessed from the sum of VF episodes over the total number of trials.

In ischaemia studies, to limit the potential for progressive effects resulting from prolonged periods of low-flow perfusion, hearts were re-perfused for 5 min between sequential measurements. Because repeat periods of low-flow may be associated with preconditioning effects, hearts were initially maximally preconditioned using 3×5-min periods of low flow, before any measurements were made.^29^ For all other studies, a minimum 1-min recovery period was allowed between subsequent pacing protocols or until measured parameters had stabilized. If VF was sustained for >30 s, hearts were cardioverted by slow injection of high KCl Krebs–Hensleit buffer (50 mmol/L) into the perfusion line. Drugs were perfused for a minimum of 10 min, or until electrophysiological parameters had stabilized for 2 min. Dynamic pacing was used to assess restitution slope (initial CL = 170 ms, 10 ms decrease every 10 beats) and S1S2 pacing was used to estimate the effective refractory period (ERP).

### 2.3 Isolated ventricular myocytes

#### 2.3.1 Transverse aortic constriction induced heart failure

Cardiac hypertrophy was induced using the transverse aortic constriction (TAC) procedure described by Kingsbury et al.^30^ but performed on animals weighing 300–450 g and with modifications to the anaesthetic regime (see Supplementary material online, Supplementary Methods, for details).

#### 2.3.2 Myocyte isolation and microelectrode recordings

Ventricular myocytes were isolated by enzymatic digestion 150 days after sham/TAC operation (for sharp electrode studies) or from adult guinea pigs (450–650 g) (for perforated patch studies) by modification of a method described in detail in Terracciano and MacLeod.^31^ Cells were superfused with normal Tyrode containing (in mmol/L); NaCl 140, KCl 6, CaCl_2_ 2, MgCl_2_ 1, glucose 10, *N*-2-hydroxyethylpiperazine-2-ethanesulphonic acid (HEPES) 10, pH adjusted to 7.4. using NaOH). Temperature was 34–36 °C. In sham and TAC, myocytes membrane potential was recorded using sharp microelectrodes (20–40 MOhm) containing (in mmol/L); KCl 2, HEPES 5, ethylene glycol-bis(2-aminoethylether)-*N*,*N*,*N*′,*N*′-tetraacetic acid (EGTA) 0.1, pH adjusted to 7.20 with KOH. Normal adult guinea pig myocytes were current-clamped in whole-cell perforated patch mode to record membrane potential. Low resistance patch pipettes were used with resistances between 1.5 and 3.0 MOhm, containing (in mmol/L); NaCl 10, KCl 23, KCH_3_O_3_ 117, MgCl 1 and HEPES 10, pH adjusted to 7.2 at 35°C with KOH. Amphotericin B was added to the pipette solution on the day of the experiment (240 µg/mL).

Restitution was assessed via a modified dynamic restitution protocol consisting of pacing for 50 beats (sharp electrodes) or 10 beats (perforated patch) at progressively shorter CLs. In a subset of studies, constant outward and inward currents were injected through the electrode to modify APD (range 20–90 pA). Bridge compensation was used to correct for series resistance errors. Additional studies investigated the influence of bretylium tosylate (10 μmol/L in H_2_O) on the dynamic restitution curve. In some studies, isolated myocytes were pre-incubated with the membrane permeable Ca chelator BAPTA-AM (100 μmol/L in DMSO).

#### 2.3.3 Ca imaging

Isolated myocytes were loaded with Fura-2-AM (5 μmol/L) for 1 h. Cells were paced at 1 Hz for 2 min before pacing at incrementally shorter CLs from 1000 to 125 ms. Intracellular Ca was measured from the ratio of emitted fluorescence (510 nm) with excitation at 340 and 380 nm (*F*_340_/*F*_380_). Systolic and diastolic Ca were normalized to stable values recorded with pacing at 1 Hz.

### 2.4 Analysis and curve fitting

Restitution curves were generated by plotting action potential duration at 90% repolarization against the preceding DI. Curves were fitted to a mono-exponential function per Eq. (1) (least squares fit). Maximum slope was measured at the minimum DI (before conduction block or VF induction) and calculated from the fit of each individual curve. Presented values represent the mean average from several experiments.

The average rate of repolarization (*R*_Repol_) was determined from the first derivative of the action potential waveform between peak and 90% repolarization (approximating a linear rate of repolarization). For mono-phasic recordings, action potential amplitude was normalized to 1.

### 2.5 Cardiac wavelength

Pseudo cardiac wavelength (λ′) was calculated for locally recorded action potentials using the method of Matthews et al.^32^ Where conduction velocity is taken as the reciprocal of activation time relative to the pacing stimulus (TAct), and the refractory period is taken as the APD at 90% repolarization. Mono-phasic action potentials were recorded from the same location throughout.

Equation 2 λ’=1/TAct*APD

### 2.6 Statistical analysis

Parametric statistical analyses were applied on the basis that the distribution of the QT interval, a surrogate of APD, is normally distributed and on the assumption that APD and measures related to APD are also normally distributed. Statistical comparisons were made using paired or unpaired Student’s *t*-tests, one- or two-way ANOVA with Tukey’s *posthoc* tests or nested general linear model ANOVA with Tukey’s *posthoc* tests, as appropriate. Categorical data were compared by Fisher’s exact tests. *P* < 0.05 was considered significant. Presented data represent mean ± SEM.

## 3. Results

### 3.1 Baseline (steady-state) APD determines the slope of the electrical restitution curve

**Figure 1:**
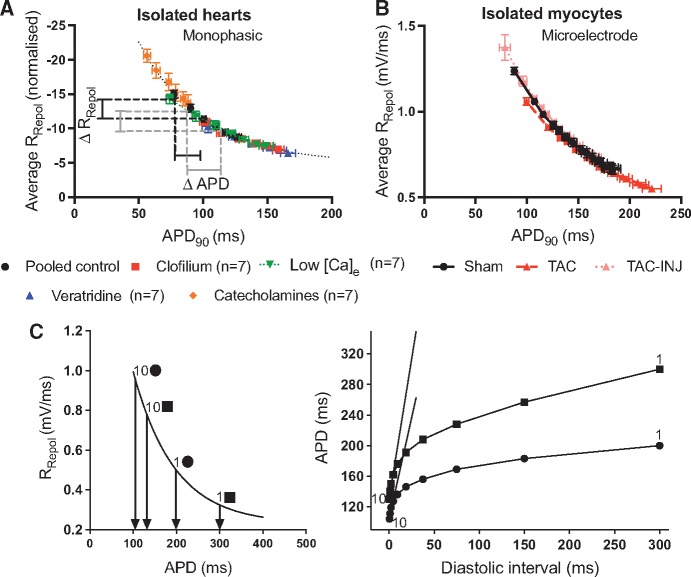
Modelling the influence of action potential duration on the electrical restitution curve. (*A*) Data demonstrating that mono-phasic action potential duration (APD_90_) is related in a non-linear manner to the average rate of repolarization (*R*_Repol_) in isolated guinea pig hearts. (*B*) The same relationship is shown in microelectrode recordings from isolated ventricular myocytes. Data represent mean ± SEM at each pacing cycle length. (*C*) The non-linear relation between the average *R*_Repol_ and APD predicts that prolongation of the action potential will act to steepen the restitution curve (see text for details). Panel C adapted with permissions from Winter and Shattock (2016).^25^

APD is determined by the rate of repolarization (*R*_Repol_) of the action potential and plotting APD as a function of the average *R*_Repol_ reveals an inverse non-linear relation, as shown in *Figure 1A* for mono-phasic action potential recordings and in *Figure 1B* for microelectrode recordings. In *Figure 1A*, data from hearts perfused with clofilium, veratridine, low [Ca]_e_ and catecholamines are shown to fall along a single mono-tonic curve. This curve represents an intrinsic property of the cardiac membrane, a consequence of geometry, as previously described.^22^^,^^25^

When the action potential is prolonged, and operating further to the right of *Figure 1A and B*, then the magnitude of change in APD for a given change in the *R*_Repol_ will be greater than that for an action potential of shorter initial duration. This is illustrated in *Figure 1A*. Therefore, in the setting of restitution, where shortening of the DI results in acceleration of repolarization rate, a proportionally greater degree of APD adaptation would be predicted for a given change in the DI. This principle is illustrated in *Figure 1C*, which shows two theoretical APD-RT curves generated from the *R*_Repol_-APD relation. Starting from a predefined APD_ss_ (circles = normal, squares = prolonged) the process of electrical restitution can be modelled as a series of incremental step increases in the *R*_Repol_, with estimated APD plotted at each step against a predefined range of Dis (note that the range of DIs is arbitrary and is the same in both curves). As such, *Figure 1C* models the acceleration of repolarization rate with decreasing DI and predicts the effect of changes in APD_ss_ on the shape of the restitution curve. *Figure 1C* predicts that restitution of APD will be of greater magnitude, and the slope of the APD-RT curve steeper, when APD_ss_ is prolonged. Importantly, steepening of the restitution curve is predicted to be independent of the specific mechanism by which APD is modified. As such, we sought to examine the role of APD in determining the slope of the restitution curve—by comparison of a range of interventions that modulate the duration of the action potential, but by different modes of action. These datasets are presented below.

### 3.2 Pharmacological and rate-dependency of APD restitution

**Figure 2:**
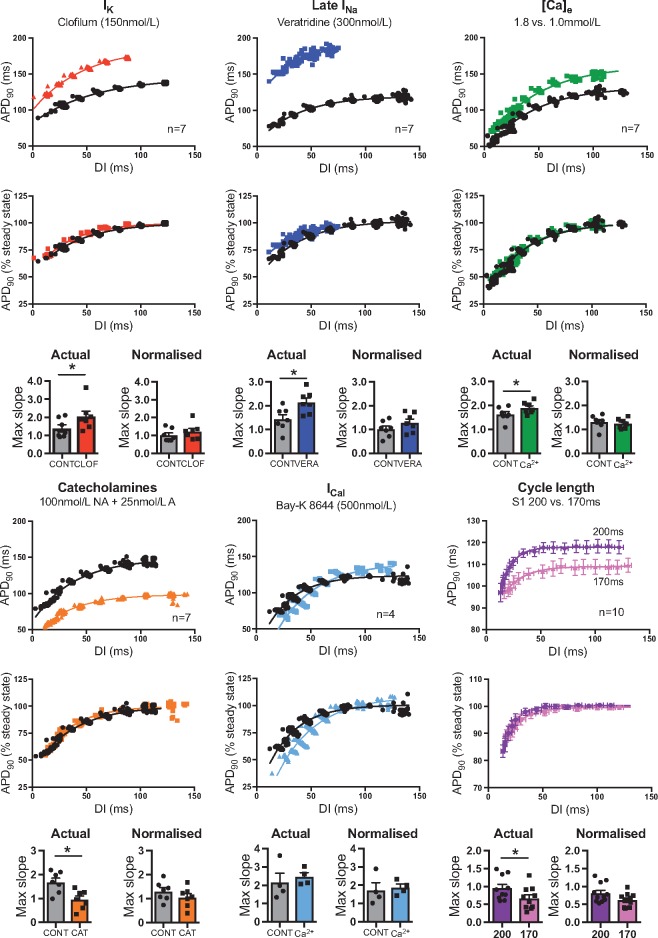
Influence of action potential duration on the kinetics of the dynamic restitution curve. Effect of electrical pacing and pharmacological interventions on the relationship between ventricular mono-phasic action potential duration (APD_90_) and diastolic interval (DI) in isolated guinea pig hearts. Top panel—actual values. Middle panel—normalized as a percentage of steady-state APD_90_. Bottom panel—mean values of maximum restitution slope for actual and normalized restitution curves. Representative curves for each experimental condition vs. control. Maximum slopes represent mean ± SEM from four to seven experiments, as noted. NA = noradrenaline. A = adrenaline. Different from control; *P < 0.05. Paired Student’s *t*-tests.

Data on the pharmacological modulation of APD and its effects on restitution curves in isolated guinea pig hearts are presented in *Figure 2*. Despite markedly different mechanisms of action, clofilium, veratridine and low [Ca]_e_ were all associated with common effects on the electrical restitution curve. Namely, that prolongation of the ventricular action potential was associated with a steepening of the dependence of APD on DI and convergence of curves at short DIs. For all interventions, the maximum slope of the dynamic restitution curve was greater when the APD_ss_ was prolonged. No change in slope was observed in hearts treated with Bay-K 8644 (±), which had no effect in APD_ss_. By comparison, the slope of the restitution curve was reduced when the ventricular action potential was shortened by catecholamine perfusion. Similar changes in restitution were also observed with rate-dependent APD-adaptation, also shown in *Figure 2*. At slower pacing rates APD_ss_ was longer and the maximum slope of the curve was greater (0.95 ± 0.11 vs. 0.67 ± 0.10, *P* < 0.05) compared to that at faster rates (i.e. 200 vs. 170 ms S1 CL). Minimum DI was comparable before and after drug treatment in all study groups (data not shown).

**Figure 3:**
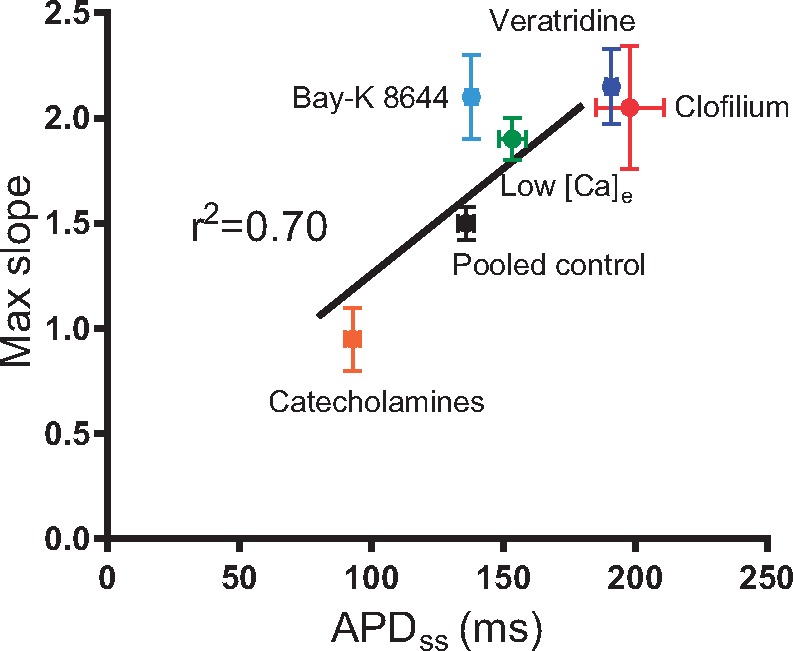
Correlation between baseline (steady-state) action potential duration and maximum slope of the dynamic restitution curve in isolated guinea pig hearts. APD_ss_=action potential duration at steady state. [Ca]_e_=extracellular calcium concentration. (*n* = 7, with the exception of Bay-K 8644 where *n* = 4) Note; Bay-K 8644 was no different from its relative control values.

Best-fit parameters derived from the mono-exponential fits of restitution curves in isolated hearts are presented in Supplementary material online, *Table S1*. No change in values of b or τ was observed in any treatment group, indicating that changes in APD_ss_ are the principal determinant altered restitution slope in all treatment groups. This is further evidenced by the fact that normalizing the restitution curves as a percentage of APD_ss_ completely abolished the effect of pacing history and pharmacological agents on the slope of the restitution curve (*Figure 2* and see Supplementary material online, *Figure S1*). *Figure 3* demonstrates a linear correlation between APD_ss_ and the maximum slope of the dynamic restitution curve; showing that APD_ss_ is an intrinsic determinant of the slope of the restitution curve, irrespective of the specific mechanism by which the action potential is modified. A comparable linear relationship was also observed in recordings from isolated ventricular myocytes (see Supplementary material online, *Figure S2*).

**Figure 4:**
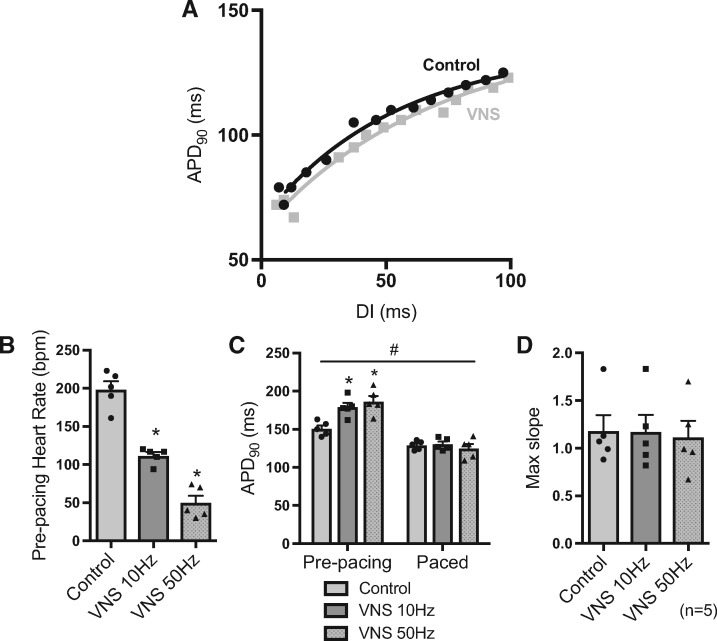
Effect of vagus nerve stimulation on the dynamic restitution curve. (*A*) Representative restitution curves under control conditions and during right vagus nerve stimulation (VNS, 10 Hz). (*B* and *C*) Mean values for intrinsic heart rate and mono-phasic action potential duration (APD_90_) in control conditions and during VNS (10 and 50 Hz). Prolongation of APD with VNS was normalized by pacing at a constant 220-ms cycle length. (*D*) Mean values for maximum restitution slope in all conditions. Data represent mean ± SEM. Different from control; *P > 0.05. The effects of pacing; ^#^*P* < 0.05. One- or two-way (repeated measures) ANOVA with Tukey’s *post hoc* tests. (*n* = 5 hearts).

### 3.3 Bretylium and vagus nerve stimulation

Previous reports suggest that bretylium, an antiarrhythmic agent, and stimulation of the cervical vagus nerve, act to flatten the slope of the restitution curve, despite prolonging ventricular APD. However, in our hands, treatment of isolated myocytes with bretylium was associated with a steepening of the slope of the restitution curve that was abolished by normalizing to APD_ss_ (see Supplementary material online, *Figure S1*). In Langedorff perfused innervated rabbit hearts, stimulation of the right cervical vagus nerve resulted in a slowing of heart rate and prolongation of ventricular APD, which was abolished by ventricular pacing (*Figure 4*). Restitution curves, compared in the absence of heart rate-dependent changes in APD, were comparable between conditions and we observed no direct effect of the vagus nerve on the slope of the restitution curve (*Figure 4*).

### 3.4 TAC induced hypertrophy

**Figure 5:**
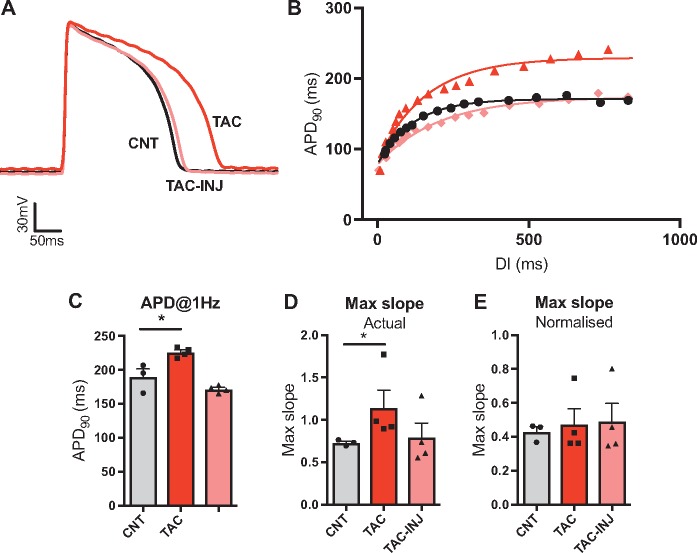
Transverse aortic constriction-induced action potential prolongation. (*A*) Representative action potential recordings (@1Hz) in isolated ventricular myocytes from animals 150-day post-transverse aortic constriction (TAC), in sham operated-controls and TAC myocytes with negative current injection (TAC-INJ) to normalize action potential duration (APD_90_). (*B*) Representative dynamic restitution curves from sham, TAC and TAC-INJ myocytes. (*C–E*) Mean data on the influence of TAC and TAC-INJ on APD_90_ (C), the maximum slope of the restitution curve (D) and maximum slope of the normalized restitution curve (E). Data represent mean ± SEM. Different from control; *P < 0.05. Nested general linear model ANOVA with Tukey’s *post hoc* tests. (sham *n* = 3 hearts with five to eight cells per heart, TAC *n*= 4 hearts with three to eight cells per heart, TAC-INJ *n* = 4 hearts with three to five cells per heart)

We also tested the hypothesis that changes in APD_ss_ are responsible for reported alterations in the restitution curve in failing hearts, using a guinea pig model of TAC-induced hypertrophy.^20^ Intracellular microelectrode recordings (using sharp electrodes) were used to compare restitution relationships in isolated ventricular myocytes from TAC and sham-operated animals. Data are presented in *Figure 5*. TAC was associated with a marked prolongation of APD (@1 Hz) when compared to sham-operated controls (*Figure 5C*), with no difference in resting membrane potential (−80.8 vs. −80.7 mV) or action potential amplitude (data not shown). Over a similar range of DIs TAC myocytes demonstrated a greater magnitude of APD shortening and an increase in maximum restitution slope. Injection of a constant negative current, sufficient to normalize APD_ss_ to sham values, abolished the increase in restitution slope associated with TAC (*Figure 5D*), indicating that changes in restitution in TAC occur secondary to prolongation of the ventricular action potential, and are not attributed to the kinetic behaviour of any ion channel or ionic conductance. As expected, outward current injection was associated with a modest reduction in the resting membrane potential (−84.2 vs. −81.3 mV, *P* < 0.05). Normalizing the restitution curve as a percentage of APD_ss_ similarly abolished the effects of TAC on the restitution curve (*Figure 5E*).

### 3.5 The influence of APD on the electrical restitution curve is preserved in the absence of intracellular calcium cycling

**Figure 6:**
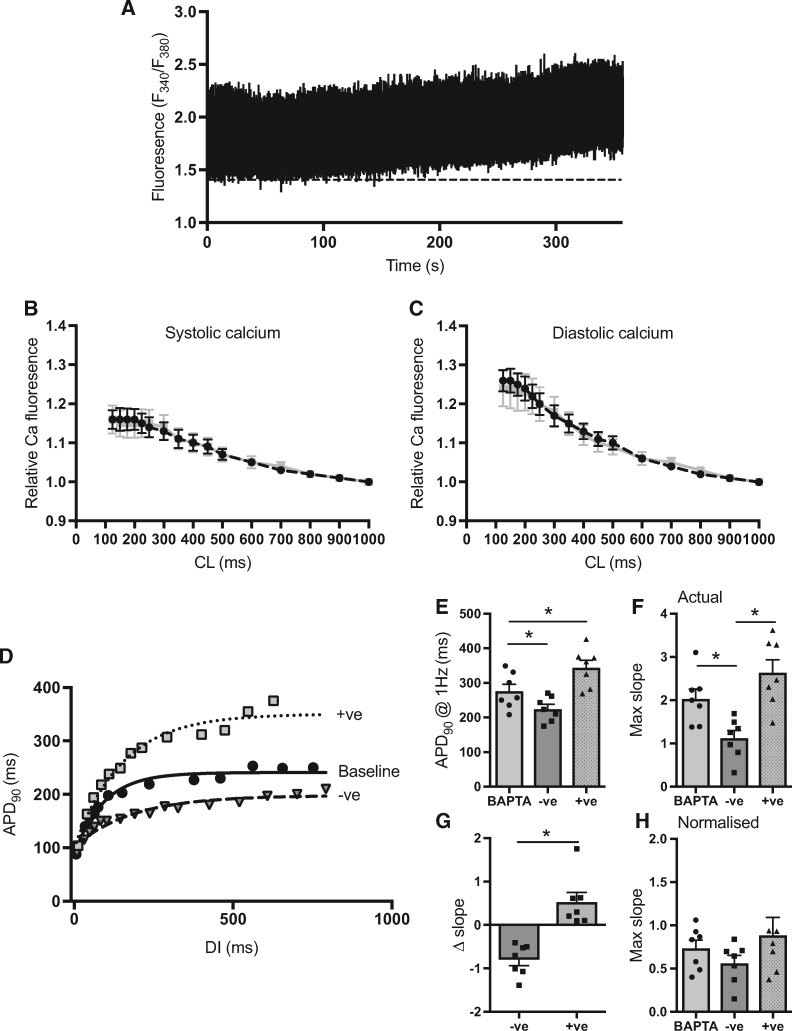
Role of intracellular calcium handling. (*A*) Representative trace demonstrating changes in calcium-dependent ratiometric Fura-2-AM fluorescence with decreasing pacing cycle length (CL). (*B* and *C*) Mean data on the relative changes in systolic and diastolic calcium fluorescence with pacing under control conditions (black) and in cells treated with clofilium (150 nmol/L, grey). (*n* = 6 hearts, 4–5 cells per heart—per condition). (*D*) Representative dynamic restitution curves from ventricular myocytes treated with the calcium chelator BAPTA-AM (100 μmol/L). Action potential duration (APD) was modified by direct current injection in perforated patch. (*E, F, G* and *H*) Mean data on the effects of outward (−ve) and inward (+ve) current injection on steady-state APD, maximum restitution slope, change in slope and steady-state APD normalised maximum slope, in BAPTA treated ventricular myocytes. Data represent mean ± SEM. Different from BAPTA; *P < 0.05. Nested general linear model ANOVA with Tukey’s *post hoc* tests or paired Student’s *t*-test. (*n* = 7 hearts, 1–3 cells per heart)

Because membrane potential and calcium handling are bi-directionally coupled, we sought to examine the role of intracellular calcium as a mediator of APD-dependent changes in restitution slope. Data on the effects of dynamic pacing on intracellular calcium concentration in isolated myocytes, as determined by Fura-2-AM fluorescence, are presented in *Figure 6*. A typical recording of the *F*_340_/*F*_380_ ratio in a single myocyte is presented in *Figure 6A* and shows an incremental increase in peak systolic and diastolic calcium with dynamic pacing. Mean data for myocytes under control conditions and with clofilium-treatment are presented in *Figure 6B and C*. No difference in the rate-dependent change of systolic and diastolic calcium was observed between groups, however, prolongation of the action potential may lead to an increase in total intracellular calcium, via increased calcium influx through *I*_Cal_. Therefore, we loaded isolated guinea pig ventricular myocytes with the membrane permeable calcium chelator BAPTA-AM (100 μmol/L), with the aim of completely abolishing the intracellular calcium-transient. APD was subsequently modulated by direct current injection of amplitude sufficient to cause a substantive change in APD_ss_ (>40 ms change, current range = 20–80 pA). Data are presented in *Figure 5D–F*. Outward (−ve) current injection led to a marked shortening, and inward (+ve) current injection a prolongation, of APD_ss_. Restitution curves measured during outward current injection were shallower than those recorded in the same cell without current injection. Conversely, positive current injection led to a steepening of the restitution curve. These data demonstrate that the influence of APD on restitution slope is independent of altered intracellular calcium handling and most likely explained by the non-linearity between APD and the *R*_Repol_ of the action potential.

### 3.6 Restitution slope does not predict vulnerability to VF

Data summarizing the effects of ibutilide, flecainide, SNS and ischaemia on ERP, restitution slope, and vulnerability to sustained VF are presented in *Table 1*. Interventions were studied in separate hearts.

**Table 1:** Susceptibility to ventricular fibrillation

	Ibutilide (*n*=7)			SNS (*n*=6)			Flecainide (*n*=6)			Ischaemia (*n*=5)		
	Cont	Treat	*P* value	Cont	Treat	*P* value	Cont	Treat	*P* value	Cont	Treat	*P* value
APD_ss_ (ms)^a^	115±2	132±3	0.010	114±5	102±3	0.045	120±2	137±3	≥0.001	123±2	86±6	0.003
ERP (ms)^a^	106±6	125±4	0.007	110±5	93±2	0.031	114±3	131±3	0.005	*	*	*
Max slope^a^	1.44±0.13	1.99±0.30	0.045	1.72±0.25	1.32±0.12	0.048	1.49±0.11	2.08±0.38	0.206	1.67±0.24	0.69±0.10	0.019
Min DI (ms)^a^	11±2	9±1	0.341	14±3	11±1	0.130	8±3	22±5	0.015	8±2	21±4	0.078
Baseline TAct (ms)	30±3	28±3	0.186	28±2	29±2	0.867	26±3	34±4	0.025	21±1	22±2	0.486
VF susceptibility												
Burst—10×30 ms^b^	0/28	0/28	>0.99	0/24	12/24	≤0.001	0/24	0/24	>0.99	3/20	12/20	0.008
Burst—60×30 ms^b^	22/28	7/28	≤0.001	18/24	20/24	0.723	17/24	0/24	≤0.001	10/20	20/20	≤0.001
**Total**^b^	22/56	7/56	0.002	18/48	32/48	<0.001	17/48	0/48	≤0.001	13/40	32/40	≤0.001

Summary data on the influence of ibutilide, sympathetic nerve stimulation (SNS), flecainide and low-flow ischaemia on action potential duration (APD), effective refractory period (ERP), dynamic restitution parameters and VF susceptibility. Colour coding: red = decrease, green = increase.

Data represent mean ± SEM, where appropriate.

^a^Paired Student’s *t*-test.

^b^Fisher’s exact test (incidence of VF/number of trials).

*P* < 0.05 are underlined.

* Accurate assessments of ERP could not be made in ischaemic conditions owing to the induction of VF at short coupling intervals.

APD_ss_, action potential duration at steady state; DI, diastolic interval; Tact, time from pacing spike to activation of action potential; VF, ventricular fibrillation.

Ibutilide is a class III anti-arrhythmic that has been reported to prolong APD and flatten the restitution curve.^26^ However, we found that ibutilide was associated with greater APD_ss_, prolonged refractoriness and a steeper restitution curve—in support of our general hypothesis. Similarly, SNS is proposed to act to shorten APD and to steepen the restitution curve.^17^^,^^27^ Our data indicate that this is not the case in the guinea pig, where SNS was associated with abbreviation of the action potential and a shallower restitution curve. The net change in the incidence of VF paralleled changes in APD_ss_ and ERP, and not steepness of restitution slope, for both interventions (see *Table 1*).

Flecainide, a class I anti-arrhythmic, acted to prolong APD_ss_ but did not significantly increase the slope of the restitution curve. This is explained by the fact that the minimum DI was shifted to the right following flecainide treatment, indicative of conduction slowing and/or post-repolarization refractoriness at short coupling intervals. That flecainide acted to slow conduction is evidenced by the increase in the time to activation of the action potential during steady-state pacing, shown in *Table 1*. Moreover, estimates of cardiac wavelength, calculated from the reciprocal of local activation time and APD, are similar to control conditions at long DIs (i.e. no net effect), but become progressively shorter, diverging from control values with decreasing DI (see Supplementary material online, *Figure S3*). This observation may be attributed to the rate-dependent action of flecainide, which increasingly slows conduction at faster rates. Despite an apparent decrease in wavelength, flecainide exerted a profound anti-arrhythmic action, suppressing susceptibility to pacing induced VF.

Data on the stability of the electrophysiological response to repeat ischaemic episodes, following maximal preconditioning, are presented in Supplementary material online, *Figure S4*. Sequential ischaemic episodes were similar in terms of the observed magnitude of action potential shortening. Short, 5 minute, episodes of ischaemia were not associated with evidence of substantial conduction slowing, with no change in the time of activation of local monophasic action potential recordings. However, ischaemia was associated with a marked flattening of the restitution curve and a dramatic increase in the susceptibility to pacing induced VF (*Table 1*). (Note: accurate assessments of ERP could not be made in ischaemic conditions owing to the induction of VF at short coupling intervals).

In summary, interventions of differing electrophysiological action exert effects on VF susceptibility that are unlikely to be attributed to changes in restitution slope, but parallel changes in APD_ss_ and refractoriness.

## 4. Discussion

In the present study, we demonstrate the importance of APD_ss_ as a determinant of the slope of the ventricular electrical restitution curve and provide evidence of a physical mechanism that relates APD to restitution kinetics, based on the seminal work of Bányász et al.^22^ The results of our study suggest that the dominant factor determining the slope of the restitution curve is that of the APD_ss_—as evidenced by the lack of effects when normalizing the curve as a function of APD_ss_. APD_ss_ and restitution slope are intrinsically related through the non-linear dependence of APD on action potential repolarization rate. Therefore, we propose a general rule that, in the absence of post-repolarization refractoriness, prolongation of the ventricular action potential will act to increase both the refractory period and the steepness of the restitution relationship. In support of this, we provide evidence of a broad range of interventions, acting through variously different mechanisms of action, but with common effects on APD and the restitution curve.

APD restitution is a fundamental process that allows adaptation of the action potential to sudden changes in rate, and has also been proposed as an important determinant of susceptibility to re-entrant arrhythmia (see later discussion). The biophysical and molecular mechanisms that underpin the shortening of the action potential with decreasing DI are well studied, and attributed to the kinetic behaviour of specific ionic currents. Over recent decades, substantial research has focussed on how differences in the expression of specific ionic currents may influence the slope of the electrical restitution curve,^19^^,^^33–35^ and also on the contribution of intracellular calcium handling to the process of action potential rate-dependent adaptation.^36^ However, these studies, and many others, have failed to account for changes in APD_ss_, which, we show, is the major determinant of the slope of the restitution curve in the ventricle. The present study provides robust evidence that changes in APD_ss/max_, and not τ or b, are the principal determinant of restitution slope in the ventricle, and, moreover, we demonstrate a simple physical mechanism that accounts for this effect—i.e. that APD is an inverse and non-linear function of the rate of repolarization of the action potential (as shown in *Figure 1*).^21^^,^^22^^,^^25^ Consequently, the magnitude of change in APD for a given change in repolarization rate is intrinsically dependent upon the APD_ss_. Zaza and Varró first proposed that this intrinsic property of the cardiac membrane, a consequence of geometry,^24^ could explain observations that drugs that modify APD commonly act in a reverse rate-dependent manner^24^, and experimental data to support this hypothesis was subsequently provided by the studies of Bányász et al. and Bárándi et al.^22^^,^^23^ These important studies focussed on steady-state action potential adaptation at relatively long CLs (300–5000 ms), where changes in APD are attributed primarily to changes in intracellular ion accumulation (and so differs from APD restitution at short DIs). Our study expands upon this idea to demonstrate the importance of non-linearity between APD and repolarization rate as a determinant of the steepness of the restitution curve, where action potential adaptation is primarily mediated by the gating properties of the sarcolemmal ion channels. Moreover, we show that the same principle applies in pathological conditions, such as heart failure. In the setting of restitution, a proportionally greater degree of APD-adaptation would be expected for a given change in DI, when the initial duration of the action potential is prolonged. Thus, the dependence of APD on DI, in the absence of some opposing action (e.g. post-repolarization refractoriness), would be predicted to be steeper. Importantly, because the non-linear *R*_Repol_-APD relation is an intrinsic mathematical property of the cardiac membrane, the influence of APD_ss_ on the restitution curve is independent of biological factors, such as specific ion channel kinetics, intracellular ionic concentrations or drug-channel binding properties. Critically, we found no evidence to support previous reports that ibutilide, bretylium and vagus nerve stimulation act to prolong APD and flatten the restitution curve.^1^^,^^27^ Indeed, our results suggest that it is highly unlikely that a drug could be specifically targeted to flatten restitution slope as a means for anti-arrhythmic therapy. However, we cannot exclude that the differences between studies reflects the species (pig vs. guinea pig for bretylium) or the pacing protocol (standard vs. dynamic restitution for vagus nerve stimulation) used. Furthermore, several studies have reported that adrenergic stimulation acts to steepen the electrical restitution curve, with experimental evidence in rabbit and in man.^17^^,^^27^ Our data suggest this is not the case in the guinea pig; a divergence that may be explained by the greater role of adrenergically stimulated *I*_Ks_ in the guinea pig and its activation on exposure to catecholamines.^37^^,^^38^

APD_ss_ is the principle determinant of restitution slope by an action that is inherently independent of the properties of any particular ion channel, exchanger or transporter, and of intracellular Ca handling. Dependency on APD_ss_ appears to explain why restitution relationships are steeper in different myocardial regions,^39^ between myocyte cell types,^19^ with drugs that prolong APD,^13–15^ and with structural/molecular remodelling in heart failure.^20^ Furthermore, flattening of the restitution curve at rapid heart rates may be explained by the fact that APD_ss_ determines the magnitude of electrical restitution and that APD_ss_ is itself a function of short-term pacing history. Thus, our study provides a framework for understanding the reported influence of short-term cardiac memory (i.e. pacing) on the slope of the restitution curve.^16^^,^^40^ The observation that the TAC phenotype could be reversed by injection of constant outward current demonstrates that the steepening of the restitution curve in pressure-overload induced cardiac hypertrophy/heart failure occurs secondary to changes in APD_ss_ and is not due to differences in the expression of ionic current(s) per se. To the best of our knowledge, this is a novel observation, however, we cannot conclude whether the same principle applies to hypertrophy induced by volume-overload. Nevertheless, it is a remarkable finding that similar changes in electrical restitution are observed with interventions of vastly differing mechanisms of action. In all cases, we observed that by normalizing the restitution curve as a percentage of APD_ss_, changes in restitution slope were abolished. Thus, with a wide array of interventions that affect outward and inward currents, changes in restitution slope appear to be principally determined by the APD_ss_. Remarkably, this remains true for class III anti-arrhythmic agents such as clofilium and bretylium, which act on *I*_Kr_ and *I*_Ks_, and appears counter to the well-described role of K^+^ channel gating in the rate-adaptation of APD, but is in keeping with previous reports.^13^^,^^34^^,^^41^^,^^42^ In principle, it might be expected that blocking *I*_Kr_ and/or *I*_Ks_ would limit APD adaptation, which would act to flatten the APD_ss_-normalized restitution curve, but we show here that this is not the case, because both clofilium, bretylium and ibutilide treatment resulted in a steepening of the restitution curve. On this basis, we tentatively conclude that the heart exhibits a remarkable ‘adaptation reserve’, wherein, loss of a single ion channel does not greatly impact the capacity of the action potential to adapt to changes in DI. This may be likened to the concept of ‘repolarization reserve’, which is proposed to limit excessive action potential prolongation and associated arrhythmogenesis.

The main finding of our study is that restitution slope and tissue refractoriness are intrinsically related, both being dependent on APD_ss_. Since refractoriness parallels APD_ss_, interventions that prolong the action potential will not only increase restitution slope but also extend the refractory period (at least at steady-state). This observation has implications for theories of arrhythmogenesis where attempts have been made to relate the induction and maintenance of VF to the steepness of the electrical restitution curve. Concomitant changes in refractoriness complicate the picture, and it is already well established that local refractoriness is a major determinant of VF maintenance.^6–8^ Broadly our data fit with this model, however, the anti-fibrillatory action of flecainide, which appears to shorten cardiac wavelength at rapid rates, clearly cannot be explained by such classical ideas. Rather, this observation is in keeping with the proposed effects of sodium channel blockade on spiral wave stability.^43^ It is tempting to speculate that concomitant changes in restitution and refractoriness may explain why the single-site restitution slope is a poor correlate for SCD. However, many other reasons have been described, including short-term cardiac memory, a lack of spatiotemporal information, and the dependence of APD alternans on intracellular calcium handling.^3^^,^^40^^,^^44^ Moreover, the studies we have described are not exhaustive and, therefore, we cannot definitively extrapolate to predict the role for APD restitution in arrhythmogenesis in all conditions, such as structural heart disease. Burst pacing, as used in the present study, generates a predictable incidence of arrhythmia and is a measure of an interventions impact on susceptibility to sustained VF. However, in the absence of high resolution spatiotemporal mapping, it is difficult to determine whether action potential prolongation is associated with greater susceptibility to re-entry (i.e. dynamic instability)—due to an increase in restitution slope—but a failure to sustain arrhythmia—due to concomitant prolongation of refractoriness. Defining the effects of slope and refractoriness on VF induction and maintenance (of which, refractoriness appears dominant in the conditions of the present studies) may be important in circumstances where cardiac remodelling results in slowing of conduction velocity, such as in the hypertrophic and failing heart. An attractive idea is that slowing of conduction velocity, in combination with action potential prolongation, could result in a scenario where dynamic instability occurs with no change, or even a decrease, in the cardiac wavelength. Thus, re-entry would be more likely to be induced and sustained. A counterpoint to this argument is that sodium channel blockade with flecainide acts to reduce susceptibility to sustained VF, despite no difference in wavelength vs. control conditions. It is, however, feasible that slowing of conduction by other mechanisms (e.g. cell hypertrophy or gap junction remodelling) may act differently to sodium channel blockade, and experiments to address this hypothesis will form the basis of future studies.

Interestingly, the relationship between APD_ss_ and restitution slope implies that greater heterogeneity of regional APD_ss_, a common consequence of heart disease, would result in greater heterogeneity of regional restitution characteristics and so generate a potentially arrhythmogenic phenotype.^11^ However, modelling studies on the influence of restitution kinetics have focussed on changes in tau, rather than APD_ss_, and our results challenge the idea that such changes occur. Studies have also implicated the restitution properties of the tissue proximal to the line of conduction block as a fundamental requirement for functional re-entry in conditions of increased dispersion of repolarization.^9^^,^^10^ Although this represents the region of tissue with shorter APD, this comparison is relative, and it is interesting to speculate that the absolute values of APD in proximal and distal regions are also an important determinant of functional re-entry. For instance, for a given difference in regional APD, the absolute values for APD may be relatively short—with flat restitution. Alternatively, the same difference in regional APD may be associated with relatively larger APDs in both regions—with steep restitution. Whether the second scenario is more likely to initiate re-entry is an interesting question for further study.

### 4.1 Study limitations

Given that our results are shown to depend upon an intrinsic property of the cardiac membrane, it is likely that any factor that prolongs the action potential would steepen the restitution curve. However, this does not preclude that the action potential could be prolonged and the restitution curve unchanged or less steep in some situations. An example of this scenario is shown in the action of flecainide, which was observed to prolong APD_ss_ but also limited the minimum DI, most likely due to post-repolarization refractoriness resulting from block of the fast inward sodium channels. In our study, we found no net effect of flecainide but it is conceivable that in the case of substantive post-repolarization refractoriness the maximum slope could be reduced. Similar effects on post-repolarization refractoriness might be expected with ischaemia, but we observed only a trend for an increase in the minimum DI, which may simply reflect the short duration of low-flow perfusion used in the present study (5 minutes). Secondly, whilst the results of this study are likely to be applicable to species other than the rabbit and guinea pig, we cannot exclude that some of interventions we have studied may exert different actions in other species because of subtle differences in ionic currents between species. Finally, we cannot conclude as to whether our results are also applicable to restitution in the atrial myocardium.

### 4.2 Conclusions

APD_ss_ is an intrinsic and important determinant of the slope of the electrical restitution curve in the guinea pig and rabbit ventricle. Prolongation of the action potential, irrespective of the underlying mechanism, would be expected to steepen the relationship between APD and DI, due to the non-linear dependence of APD on repolarization rate. Concomitant changes in restitution slope and refractoriness complicate theories on the mechanisms of re-entrant arrhythmia that consider for only one factor or the other. In the conditions we have studied, VF susceptibility appears to be predominantly determined by changes in refractoriness, rather than restitution slope. Dependence on APD_ss_ may contribute to the failure of single-site restitution slope to predict sudden cardiac death.

## Supplementary material

Supplementary material is available at *Cardiovascular Research* online.

## Supplementary Material

Supplementary Data
