# Supplementary material for: Restitution slope is principally determined by steady-state action potential duration
Source: Cardiovasc Res. 2017 Mar 23;113(7):817–28. doi: 10.1093/cvr/cvx063 (PMC5437364; doi:10.1093/cvr/cvx063)
Supplement: Supplementary Data [file cvx063_Supp.docx]

**Supplementary Methods**

Transverse aortic constriction induced heart failure

Dunkin-Hartley guinea pigs were individually housed at 21 ± 1^o^C on a 12-hour light:dark cycle and provided with feed and water ad libitum. Cardiac hypertrophy was induced using the transverse aortic constriction (TAC) procedure described by Kingsbury *et al.*^30^ but performed on animals weighing 300–450g and with modifications to the anesthetic regime. Anesthesia was induced with a 4% isoflurane in 100% O_2_ (Isoflurane-Vet, Merial). Once anaesthetized animals were administered atropine sulphate (0.05 mg.kg-1 i.m., Atrocare, Animalcare Limited) to reduce the oral and nasal secretions and facilitate endotracheal intubation.  Lidocaine (Xylocaine 10mg, AstraZeneca) spray was applied prior to endotracheal intubation with a 14G intravenous catheter (length 6.4mm). After intubation, anaesthesia was maintained with 1.5~2% isoflurane in oxygen via the catheter. Animals were ventilated with a Zoovent Mini ventilator (Triumph Technical Services, Milton Keynes, UK) at 70ml/kg and 70cycles/min. End tidal CO2, peripheral O2 saturation and pulse rate were monitored with a Capnovet-10 (Vetronic Services, Devon, UK) and pulse-oxymeter (SurgiVet, Smiths Medical, Dublin, OH, U.S.A). Prophylactic antibiotics (enrofloxacin (Baytril, Bayer) 5mg/kg s.c.) and analgesia (carprofen (Rimadyl, Pfizer) 4.4 mg/kg s.c.) were given after a stable anesthesia had been achieved. Local anesthetic was administered (bupivacaine (Marcain 0.25%, AstraZeneca, UK) by subcutaneous injection around the incision site. Carprofen (Rimadyl, 20 mg in 400ml water) was given orally for 4 days to maintain post-operative analgesia. Sham animals underwent all procedures excluding TAC.


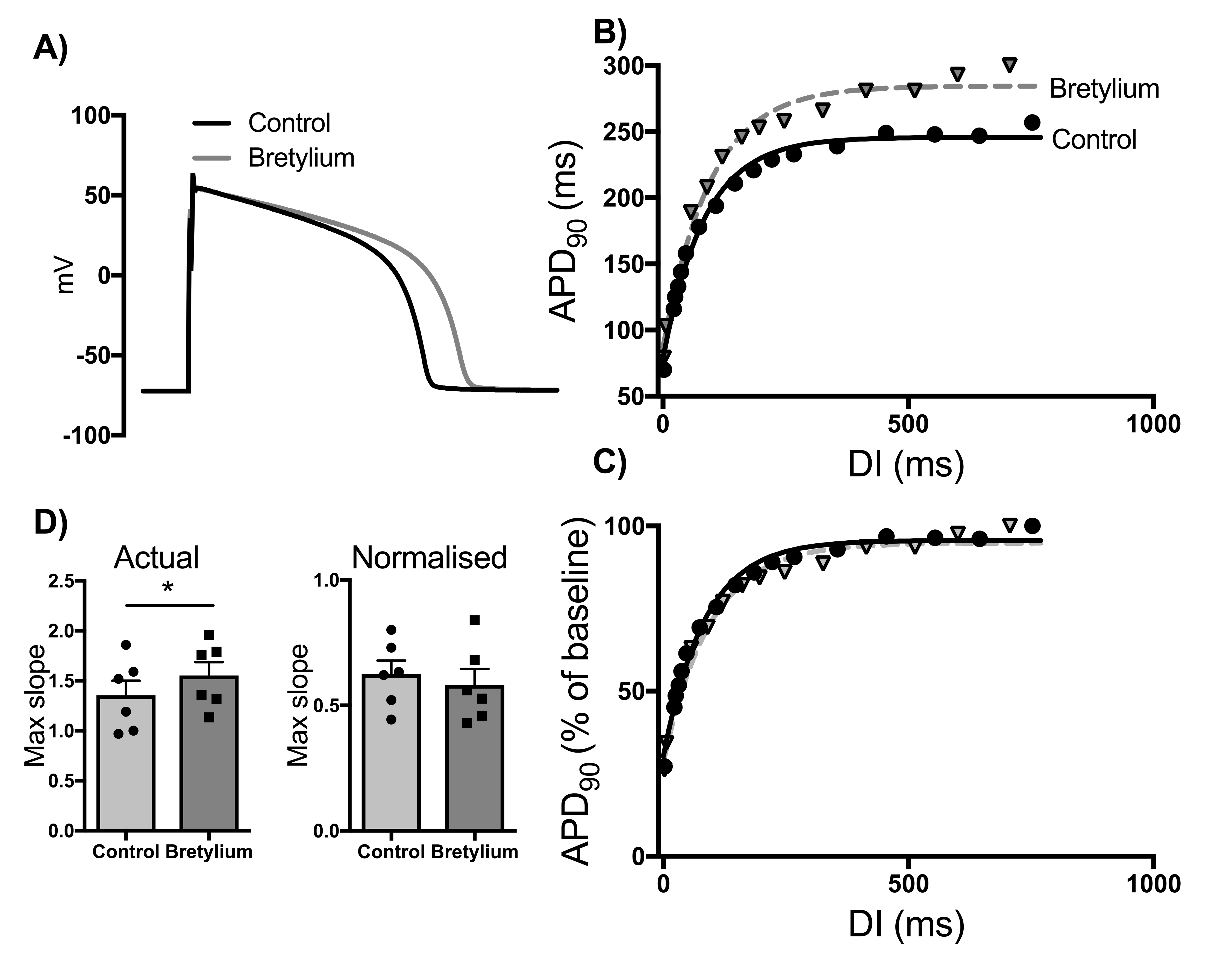


**Supplementary Figure 1. Influence of bretylium on the electrical restitution curve. A)** Representative recordings of action potentials in an isolated guinea pig myocte before and after perfusion of bretylium (10μmol/L). **B)** Example restitution curves measured under control conditions and after bretylium perfusion. **C)** The same curves normalised as a function of the baseline (steady-state) action potential duration (APD_90_). **D)** Mean values for the maximum slope of the restitution curve before (absolute) and after (relative) normalisation to steady-state APD. Data represent mean ± SEM. Different from control; *p<0.05. Paired Student’s t-tests. (n=6 hearts, 1-3 cells per heart)


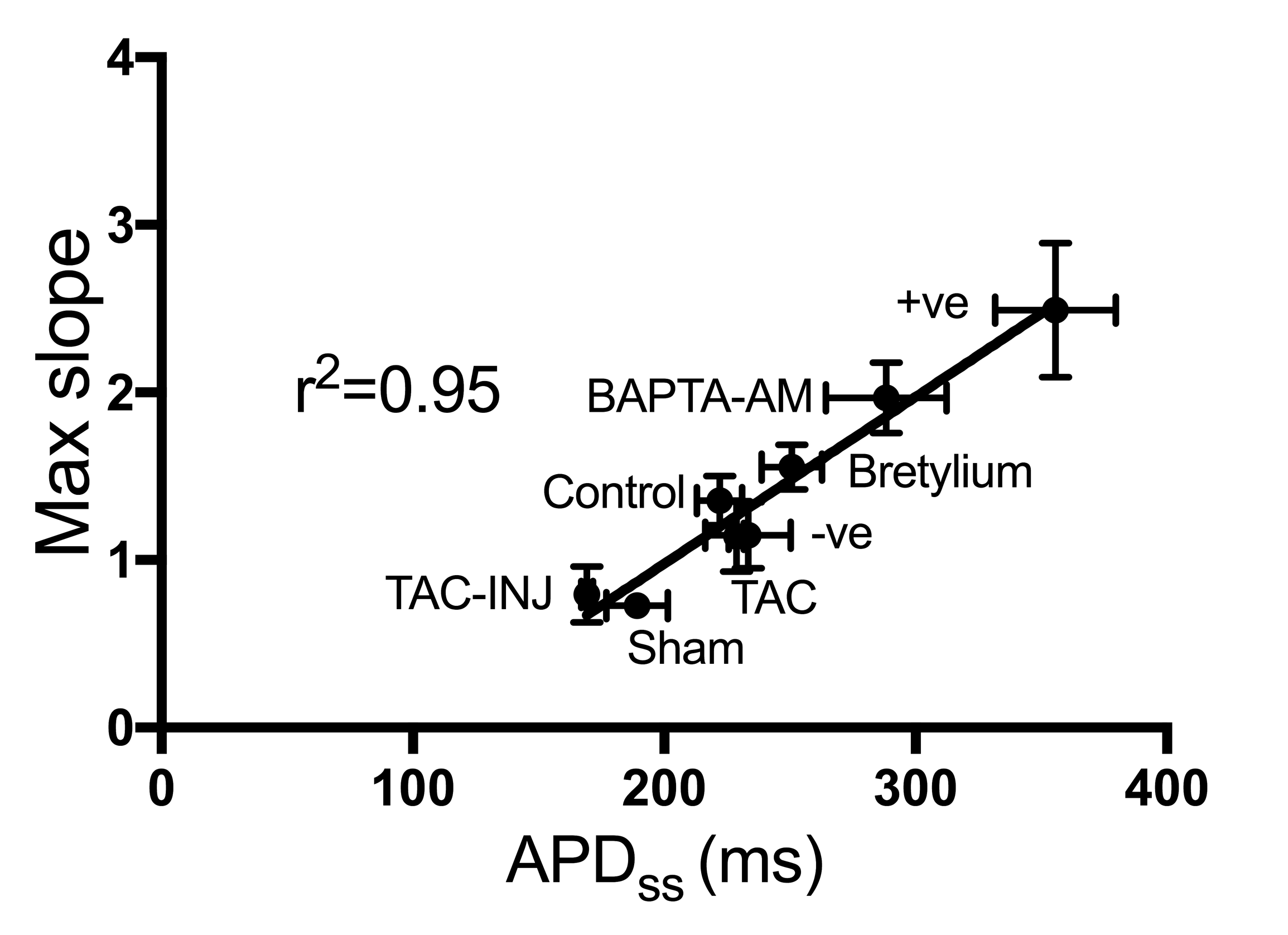


**Supplementary Figure 2. Linear correlation between steady-state action potential duration and the maximum slope of the restitution curve in isolated guinea pig ventricular myocytes.** Data from sharp microelectrode and perforated patch experiments. LEGEND: –ve=BAPTA-AM treated cells with outward current injection; +ve=BAPTA-AM treated cells with inward current injection; Control=control data for bretylium experiments; TAC=transverse aortic constriction; TAC-INJ=transverse aortic constriction with outward current injection; Sham=sham for transverse aortic constriction experiments; APD_ss_=action potential duration at steady-state. Data represent mean ± SEM

**
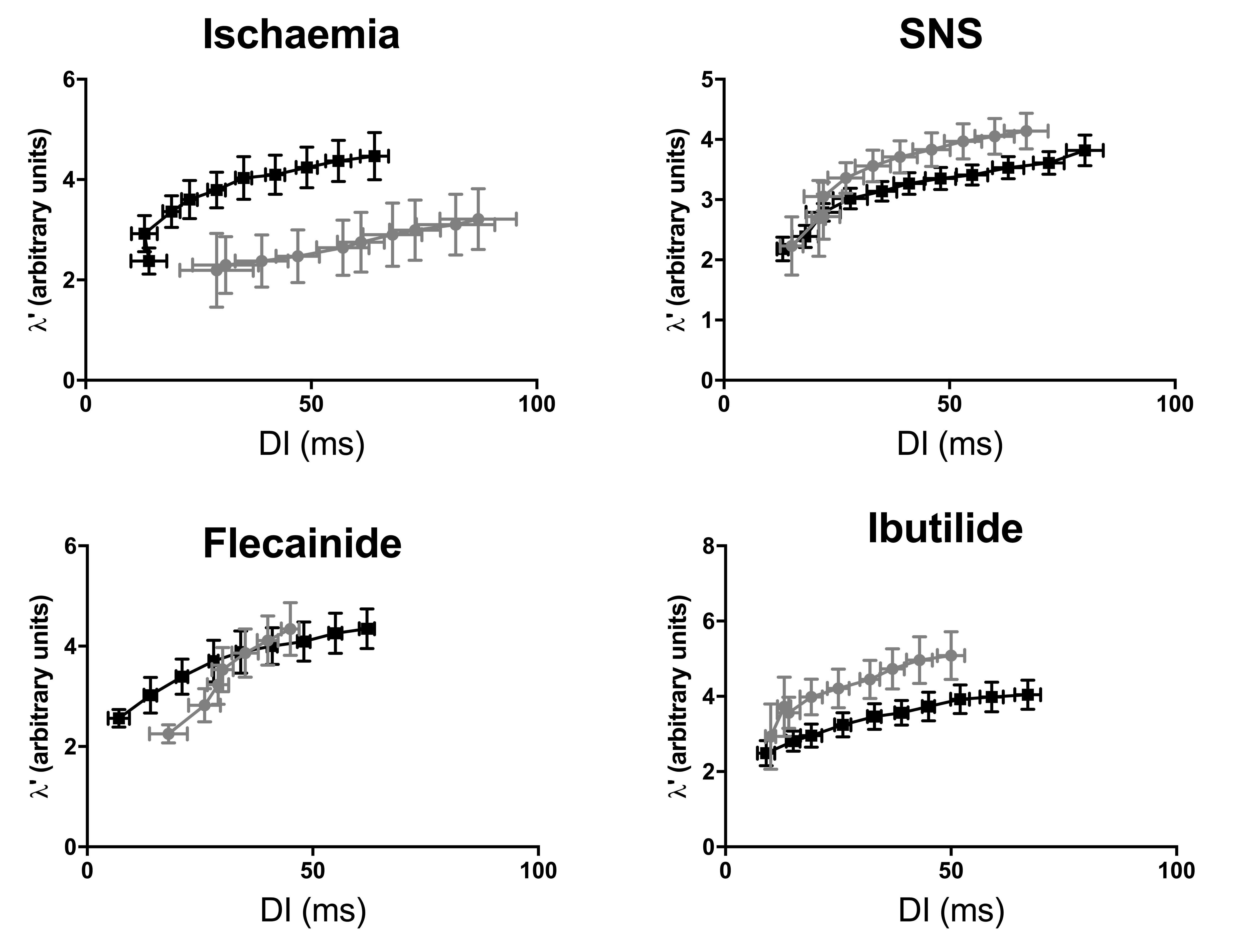
**

**Supplementary Figure 3. Estimates of cardiac wavelength.** Pseudo cardiac wavelength as a function of diastolic interval in low-flow global ischaemia, with sympathetic nerve stimulation (SNS), and following treatment with flecainide and ibutilide. Data represent mean ± SEM. (n=5-7 hearts)

**
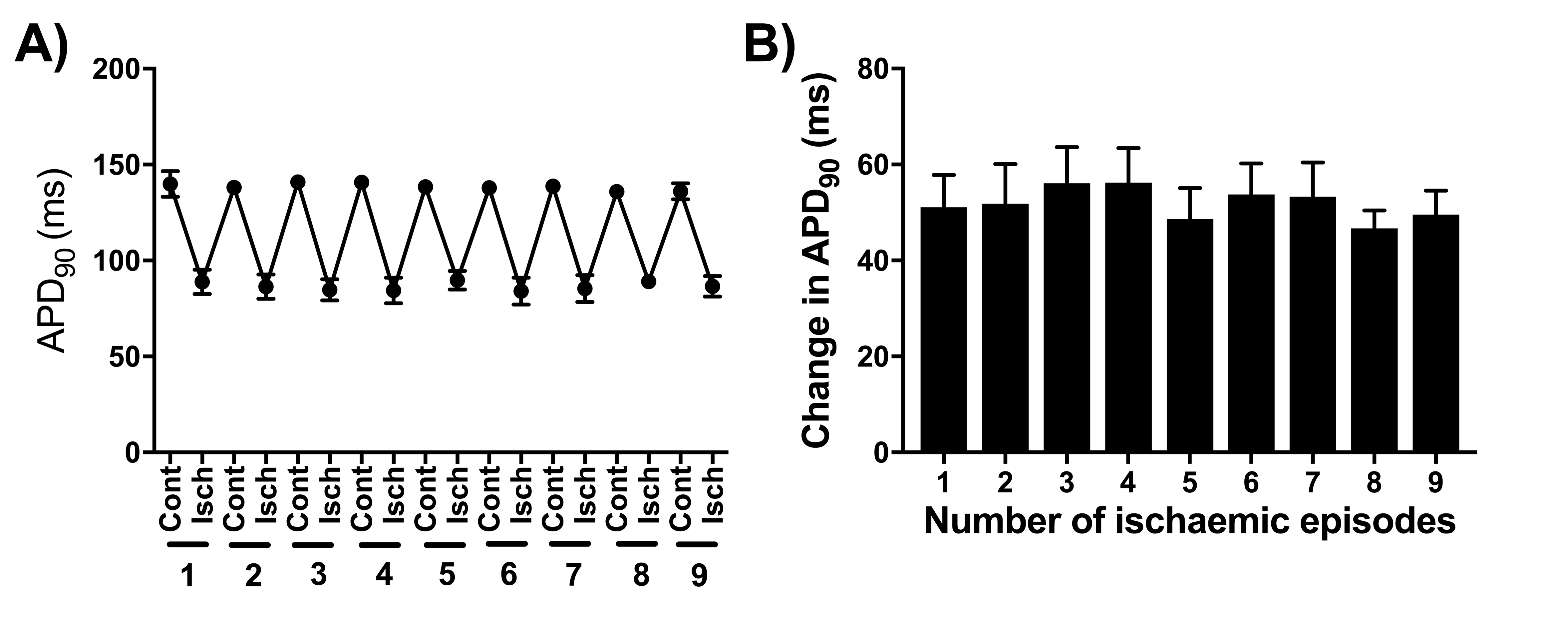
**

**Supplementary Figure 4. Reproducibility of electrophysiological responses to repeat ischaemic episodes.** A) Data represent values of action potential duration (APD_90_) at in control (Cont) conditions and following 5-minutes of low-flow ischaemia (Isch). Hearts were paced at 5Hz and were reperfused for 5-minutes between subsequent ischaemic episodes. Numbers denote the number of ischaemic episodes. B) Mean data demonstrating the change in APD_90_ with subsequent ischaemic episodes. Data represent mean ± SEM. (n=5 hearts)

**Supplementary Table 1. Best-fit parameters for restitution curves in isolated hearts**

|  | **MAPD_90_max (ms)** | | | **τ** | | | **b** | | | **Max slope** | | |
| --- | --- | --- | --- | --- | --- | --- | --- | --- | --- | --- | --- | --- |
|  | **Cont** | **Inter** | **p value** | **Cont** | **Inter** | **p value** | **Cont** | **Inter** | **p value** | **Cont** | **Inter** | **P value** |
| **Clofilium (100nmol/L) (n=7)** | 145±5 | 198±13 | <0.001 | 54±7 | 52±7 | 0.571 | 0.56±  0.04 | 0.63±  0.05 | 0.304 | 1.4±0.2 | 2.1±0.3 | 0.020 |
| **Veratridine (300nmol/L) (n=7)** | 134±7 | 191±19 | 0.004 | 42±3 | 36±5 | 0.288 | 0.59±  0.04 | 0.49±  0.04 | 0.076 | 1.4±0.2 | 2.1±0.2 | 0.028 |
| **Low [Ca]_e_ (1.8 vs 1.0mmol/L) (n=7)** | 132±6 | 174±11 | 0.012 | 43±5 | 54±4 | 0.185 | 0.72±  0.06 | 0.64±  0.04 | 0.196 | 1.6±0.1 | 1.9±0.1 | 0.031 |
| **Catecholamines (100nmol/L noradrenaline + 25nmol/L adrenaline) (n=7)** | 141±10 | 93±3 | <0.001 | 48±9 | 46±12 | 0.704 | 0.62±  0.02 | 0.56±  0.08 | 0.416 | 1.7±0.2 | 1.0±0.2 | <0.001 |
| **Bay-K 8644 (500nmol/L) (n=4)** | 127±2 | 137±4 | 0.055 | 36±5 | 41±7 | 0.266 | 0.59±  0.04 | 0.49±  0.04 | 0.076 | 1.9±0.3 | 2.1±0.2 | 0.628 |
| **S1 pacing rate (200 vs. 170ms CL) (n=10)** | 118±3 | 109±4 | <0.001 | 15±2 | 18±5 | 0.448 | 1.15±  0.18 | 1.12±  0.58 | 0.958 | 1.0±0.1 | 0.7±0.1 | 0.042 |

Cont=Control, Inter=Intervention, [Ca]_e_=extracellular calcium, CL=cycle length. p<0.05 are underlined.
